# Acetaldehyde and parkinsonism: role of CYP450 2E1

**DOI:** 10.3389/fnbeh.2013.00071

**Published:** 2013-06-21

**Authors:** Francesca Vaglini, Cristina Viaggi, Valentina Piro, Carla Pardini, Claudio Gerace, Marco Scarselli, Giovanni Umberto Corsini

**Affiliations:** Department of Translational Research and New Technology in Medicine, University of PisaPisa, Italy

**Keywords:** CYP450 2E1 isozyme, acetaldehyde, Parkinson's disease, dopaminergic neurons, ethanol

## Abstract

The present review update the relationship between acetaldehyde (ACE) and parkinsonism with a specific focus on the role of P450 system and CYP 2E1 isozyme particularly. We have indicated that ACE is able to enhance the parkinsonism induced in mice by 1-methyl-4-phenyl-1,2,3,6-tetrahydropyridine, a neurotoxin able to damage the nigrostriatal dopaminergic pathway. Similarly diethyldithiocarbamate, the main metabolite of disulfiram, a drug widely used to control alcoholism, diallylsulfide (DAS) and phenylisothiocyanate also markedly enhance the toxin-related parkinsonism. All these compounds are substrate/inhibitors of CYP450 2E1 isozyme. The presence of CYP 2E1 has been detected in the dopamine (DA) neurons of rodent Substantia Nigra (SN), but a precise function of the enzyme has not been elucidated yet. By treating CYP 2E1 knockout (KO) mice with the neurotoxin 1-methyl-4-phenyl-1,2,3,6-tetrahydropyridine, the SN induced lesion was significantly reduced when compared with the lesion observed in wild-type animals. Several *in vivo* and *in vitro* studies led to the conclusion that CYP 2E1 may enhance the 1-methyl-4-phenyl-1,2,3,6-tetrahydropyridine toxicity in mice by increasing free radical production inside the dopaminergic neurons. ACE is a good substrate for CYP 2E1 enzyme as the other substrate-inhibitors and by this way may facilitate the susceptibility of dopaminergic neurons to toxic events. The literature suggests that ethanol and/or disulfiram may be responsible for toxic parkinsonism in human and it indicates that basal ganglia are the major targets of disulfiram toxicity. A very recent study reports that there are a decreased methylation of the CYP 2E1 gene and increased expression of CYP 2E1 mRNA in Parkinson's disease (PD) patient brains. This study suggests that epigenetic variants of this cytochrome contribute to the susceptibility, thus confirming multiples lines of evidence which indicate a link between environmental toxins and PD.

## Alcohol consumption and Parkinson's disease

A large number of case-control studies analysed the relationship between Parkinson's disease (PD) and some environmental factors (De Lau et al., 2004). Addictive behaviors, such as cigarette smoking and coffee drinking, showed a protective effect against PD and parkinsonism. Regarding alcohol consumption, most studies report either a moderately decreased risk or no change in risk associated with alcohol intake (Benedetti et al., 2000; Paganini-Hill, 2001; Hernan et al., 2004; Wirdefeldt et al., 2011; Campdelacreu, 2012; Noyce et al., 2012; Palacios et al., 2012). In contrast with these clinical findings, experimental data in rodents showed that alcohol induces a reduction in the dopamine (DA) levels in the midbrain, even if contradictory data are present in literature and an increased oxidative stress in nigral cells (Collins, 2002; Ambhore et al., 2012) and Golgi fragmentation (Tomas et al., 2012). Likely, alcohol consumption and neurodegenerative disease (e.g., PD) induce similar effects on intracellular structures and trafficking (Ambhore et al., 2012). It's conceivable that some components of alcoholic beverages (e.g., flavonoids in red wine) could have a neuroprotective activity (Palacios et al., 2012). Beer drinkers have a lower risk of PD (Hernan et al., 2003), and this result can be explained with higher plasma urate levels that beer induces. Indeed, urate plays a protective role against PD (Xiang et al., 2008). It has been known that in parkinsonian patients addiction such as cigarette smoking, drug consumption, alcoholism and compulsive disorders such as gambling, compulsive shopping, hypersexuality are less common than into general population (De Lau et al., 2004; Xiang et al., 2008; Noyce et al., 2012). Data collected in the last decades all over the world highlight the link between dopaminergic replacement and onset of addiction behaviors and compulsive disorders in Parkinson's patients (Villa et al., 2011). However, alcohol consumption and a history of alcoholism seem to be related with higher incidence of impulse control disorders in PD patients receiving dopaminergic replacement (Evans et al., 2005; Wu et al., 2009). Some endogenous alkaloids, like salsolinol (1-methyl-6,7-dihydroxy-1,2,3,4-tetrahydroisoquinoline,SAL) and tetrahydropapaveroline (6,7-dihydroxy-1-(3′,4′-dihydroxybenzyl)-1,2,3,4-tetrahydro-isoquinoline; THP), whose blood levels are increased by alcohol, have neurotoxic effects especially in the striatum and reduce DA content into basal ganglia (Young-Joon and Hyun-Jung, 2010). THP is detected at a high level in the urine of parkinsonian patients under L-DOPA therapy (Cashaw, 1993); however, there are very low levels of THP in the urine of abstinent alcoholics. Possible implications of THP in alcohol dependence were inferred from the observation that rats which normally rejected alcohol, would drink alcohol in excessive amount following direct delivery of THP (Duncan and Deitrich, 1980). Thus, these substances might contribute to alcohol dependence (Collins, 2004; Hipólito et al., 2012; Deehan et al., 2013). These factors can be useful to identify PD patients at high risk of developing impulse control disorders during dopaminergic replacement.

## Acetaldehyde and Parkinson's disease

**Figure 1:**
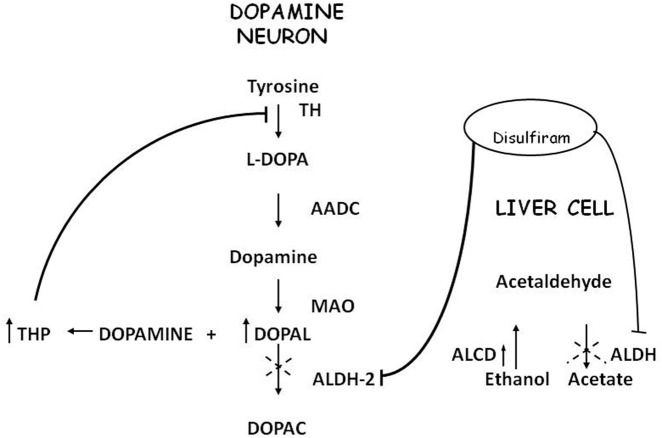
**Convergence of metabolic pathway for acetaldehyde and dopamine**.

Acetaldehyde (ACE) is the alcohol metabolite responsible for unpleasant effects such as nausea, vomiting, tachycardia and hypotension. ACE increases the toxic effect of 1-methyl-4-phenyl-1,2,3,6-tetrahydropyridine (MPTP) in striatum (Corsini et al., 1987; Zuddas et al., 1989a,b, 1992; Vaglini et al., 1996); this activity has been demonstrated in mice but not in rats (Fornai et al., 1997). There are no studies in humans that relate ACE to PD or parkinsonism, most likely because it's not a substance of abuse nor a drug nor a pollutant. ACE is further metabolized to acetic acid with catalysis of Aldehyde dehydrogenase (ALDH); disulfiram, a blocker of this enzyme, induces ACE accumulation and is used in treatment of alcoholism. Disulfiram can have a neurotoxic effect; namely, acute intoxication induces unilateral pallidal lesion as described by means of magnetic resonance evaluation and clinical assessment (De Mari et al., 1993) while chronic administration can induce Wernicke's encephalopathy, with progressive frontal decline and akineto-rigid parkinsonism (Charles et al., 2006). Brain MRI revealed symmetrical and reversible lesions in basal ganglia, but after discontinuation of disulfiram, clinical recovery was slow and partial. These observations suggest that basal ganglia are the major targets of disulfiram neurotoxicity (Laplane et al., 1992). Similar brain lesions are also observed in “energy deprivation syndromes,” which are toxic, genetic or nutritional disorders that disrupt enzymes involved in energy production pathways (Laplane et al., 1992; Charles et al., 2006). Probably, disulfiram impairs cellular processes involved in ATP production but the exact mechanism remains unclear. It's conceivable that disulfiram toxicity is linked at least in part to the action of ACE; indeed other aldehydes, such as 3,4-dihydroxyphenilacetaldehyde (DOPALD), an oxidative metabolite of DA, have been shown to have a neurotoxic activity (Gesi et al., 2001) and higher levels of these compounds are related with neurodegenerative diseases (Marchitti et al., 2007) (Figure 1).

## Acetaldehyde and experimental parkinsonism

**Figure 2:**
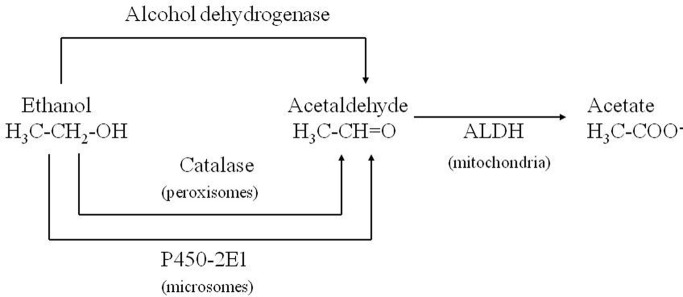
**Role of CYP 2E1 in metabolizing ethanol and acetaldehyde**.

Corsini et al. (1985) unexpectedly found that diethyldithiocarbamate (DDC), the main metabolite of disulfiram, markedly enhanced the MPTP-induced parkinsonism in mice. This effect was initially interpreted as due to the inhibition of superoxide dismutase leading to an increase in oxidative stress induced by the toxin. Subsequently, among numerous compounds tested, other enhancers of MPTP toxicity (ethanol and ACE) were found by the same authors (Corsini et al., 1987). After this further discovery, this group suggested that these compounds could increase the potency of the toxin via an inhibition of ALDH within the striatum. The “enhancers,” at the same time, prolonged the striatal half-life of 1-methyl-4-phenylpyridinium ion (MPP^+^), the toxic metabolite of MPTP, (Irwin et al., 1987; Zuddas et al., 1989b) and this was interpreted as the causative factor of this enhancement. However, in 1996 an article by Vaglini et al. demonstrated that striatal MPP^+^ levels do not necessarily correlate with MPTP toxicity in the same animal species (mouse) and they further on suggested, as previously reported, that DDC-increased toxicity was probably due to an independent action on glutamate receptors (Vaglini et al., 1996). However it is likely that the prolonged storage of MPP^+^ inside the DA neurons was crucial for its toxic effects. According to this interpretation, the enzymes, which may determine the disposition of MPP^+^ inside the DA neurons, have a cardinal role in MPTP toxicity. CYP 2E1 and the CYP 2D family are the most widely represented isozymes within the DA neurons (Watts et al., 1998; Riedl et al., 1999) and it is likely that these two P450 enzymes are responsible for MPP^+^ clearance. As a matter of fact, DDC, ethanol and ACE have been discovered to be specific substrates/inhibitors of CYP 2E1 when they are acutely administered (Stott et al., 1997) (Figure 2). This specific inhibition inside the DA neuron may account for the increase in MPP^+^ striatal half-life, and thus toxicity.

More recently, furthermore, we demonstrated that, similar to DDC and ACE, CYP 2E1 substrates/inhibitors, such as diallylsulfide (DAS) or phenylethylisothiocyanate (PIC) markedly enhance MPTP toxicity in C57/bl brain, suggesting an involvement of CYP 2E1 in the enhancement of MPTP toxicity. However, because DAS and PIC are not true CYP 2E1 inhibitors (Nissbrandt et al., 2001), in order to provide direct evidence for CYP 2E1 involvement, CYP 2E1 knockout (KO) mice and their wild-type counterparts were challenged with the combined treatment DDC+MPTP (Vaglini et al., 2004). In that article we consistently showed that an inhibition of the enzyme, as obtained with DDC challenge, failed to enhance the toxic effect of MPTP in CYP 2E1 KO mice, whereas the effect was regularly present in wild-type animals. Focusing on this, we have studied the sensitivity of CYP 2E1 KO mice to the toxin (Viaggi et al., 2009). The lack of CYP2 E1 did not increase MPTP toxicity as expected from previous experiments with the inhibitors, and, in fact, the CYP 2E1 KO mice showed a significant resistance to DA neuronal lesions induced by the toxin in comparison with their wild-type counterparts. The reduced sensitivity to MPTP of CYP 2E1 KO mice, but not the complete insensitivity, may be due to compensatory mechanisms taking place because of the missing protein. This phenomenon was observed very clearly by Gonzalez when his team generated these mice in order to study acetaminophen-induced liver toxicity (Lee et al., 1996). This drug causes liver and kidney necrosis when it is metabolized to an alkylating intermediate by the P450 system, and more specifically by CYP 2E1 (Jollow et al., 1973; Mitchell et al., 1973; Gonzalez, 2007). CYP 2E1 KO mice were generated to strengthen the specific role of CYP 2E1 during acetaminophen toxicity (Lee et al., 1996). The CYP 2E1 KO mice were less sensitive to the hepatotoxicity of the drug but they were not completely unaffected. A compensatory isozyme of the P450, probably CYP2D6, was substituted, thus producing the same—though reduced—toxic effect. Very recently we generated mesencephalic cell cultures from CYP 2E1 KO and wild-type embryos to investigate MPP^+^ toxicity and its cell distribution. In this model we demonstrated that a trace amount of MPP^+^ accumulates inside the neurons from KO mesencephalic cultures in a quantity double than that from wild-type embryos, although the KO cultures are less lesioned by the toxin (manuscript in preparation). We then suggested, once inside the cell (or striatal synaptic terminal), MPP^+^ entered preferentially into vesicles where its storage represented a sort of protection with respect to other toxic sites such as mitochondria.

In conclusion CYP 2E1 may facilitate the transfer of MPP^+^ to mitochondria for further metabolism. Alternatively and independently from MPP^+^ disposition, CYP 2E1 produces toxic reactive intermediates from endogenous or exogenous substrates which in turn impair neuronal viability.

## The P450 system in PD

In 1985, Barbeau et al. elegantly presented evidence for an association of a CYP 2D6 defect with PD (Barbeau et al., 1985). Indeed, they postulated that subjects with a reduced CYP 2D6 enzyme (poor metabolizers) are vulnerable for PD because of the impaired capacity of liver to detoxify those neurotoxins which are harmful for DA neurons. It is worth noting that further studies have actually indicated CYP 2D6 as the major detoxifying liver enzyme for the PD-inducing neurotoxin, MPTP (Coleman et al., 1996; Gilham et al., 1997). After this pioneering report, however, many conflicting results were obtained in phenotypic and genotypic CYP 2D6 studies, which have been outlined in a comprehensive review by Riedl et al. (1998).

Several enzymes involved in the metabolism of endogenous compounds and xenobiotics have been studied in relation to PD. However, CYP450 in particular drew attention, due to its ability to defend the body against xenobiotic aggression. In particular, six P450 enzymes have been examined with respect to PD: CYP 1A1 (Kurth and Kurth, 1993; Bennet et al., 1994; Takakubo et al., 1996), CYP 2C9 (Ferrari et al., 1990; Peeters et al., 1994), CYP 2C19 (Gudjonsson et al., 1990; Tsuneoka et al., 1996), CYP 1A2, CYP 2E1 (Factor et al., 1989; Steventon et al., 1989), and CYP 2D6 (Riedl et al., 1998). Since the first enthusiastic claim, more than 50 reports have debated the role of CYP 2D6 in the pathogenesis of PD. Subsequent phenotypic studies have failed to support a link between this isozyme and PD. Similarly, the most extensive genetic studies initially confirmed this link, but a critical analysis of the recent studies from different groups again failed to draw any definitive conclusion (Riedl et al., 1998). Indeed, with respect to CYP 2D6, no laboratories have succeeded in replicating the initial report of Smith et al. (1992), according to which the frequency of poor metabolizers significantly increased in a PD population. Subsequent reports have been conflicting, although some groups have claimed differences in the allelic frequency of CYP 2D6^*^4 and other CYP 2D6 allelic variants in PD. Two recent meta-analyses failed to find an increased frequency of poor metabolizers among PD patients (Christensen et al., 1998; Rostami-Hodjegan et al., 1998). On the contrary, an earlier meta-analysis suggested a weak association, but this included fewer studies (McCann et al., 1997). As a result of their inability to observe any association, other authors performed sub-group analyses, thus suggesting a possible link with “young onset PD” (Agundez et al., 1995) or PD with prominent tremor (Akhmedova et al., 1995). Unfortunately, these findings have not been replicated, either (Sandy et al., 1996). Although most studies have been negative, there are some critical issues that have been addressed by Le Couteur and McCann (1998) in connection with this problem. First, it is unlikely, on the basis of current studies, to completely refute the involvement of CYP 2D6, as, in order to have a definitive study of a statistical power, one would need almost 3000 subjects to exclude a 50% increase in the frequency of poor metabolizers among PD patients. The second issue is that studies should consider only patients who have had neurotoxin exposure. If CYP 2D6 polymorphism influences vulnerability to PD by affecting the metabolism of an environmental neurotoxin, then studies should include only those subjects who have undergone this kind of neurotoxin exposure. The authors concluded that this stratification for toxin exposure is necessary in order to rule out the role of CYP 2D6 in the pathogenesis of PD.

This last concept of an environmental toxin and CYP 2D6, as its metabolizing enzyme, opens an old issue regarding the toxic hypothesis of PD, which originated from the incidental discovery of MPTP as a widespread impurity (Langston et al., 1983). Indeed, MPTP is metabolized by some P450 enzymes and by CYP 2D6 in particular (Coleman et al., 1996; Gilham et al., 1997) and has recently been discovered to be a synthetic impurity of heterocyclic drugs (Kramer et al., 1998). In this study, the authors assessed the risk of administering MPTP orally and reported that compounds containing less than 5 p.p.m. of MPTP do not involve any neurotoxicological health risk. They concluded surprisingly that it may be assumed that MPTP is also present as a yet undiscovered minor impurity in various existing drugs (Kramer et al., 1998). If this was true, MPTP or one of its analogues would represent the toxin probably responsible not for idiopathic PD, but for a specific subgroup of parkinsonism. In this case, CYP 2D6-related metabolism would be of extreme importance and phenotypic and genotypic studies should be carried out on different and selected types of subjects.

## P450 in experimental parkinsonism

In general, MPP^+^ metabolism, unlike MPTP, has been poorly investigated. Johannessen et al. (1985) postulated that MPP^+^ may be transformed into free radical species, and other authors provided evidence for CYP 2D isoform involvement (Fonne-Pfister and Meyer, 1988; Jolivalt et al., 1995). It is interesting to note that CYP 2E1 is associated with the metabolism of several small planar molecules, such as nitrosoamines, benzene, alcohol and 3-hydroxypiridine (Parkinson, 1996), and is present in a functional form because its levels can be induced by prior treatment with isoniazid (Park et al., 1993). CYP 2E1 therefore may represent, in this particular case, a detoxification pathway of MPP^+^, whose inhibition by DDC leads to an increased toxicity. A similar conclusion can be drawn for CYP 2D isozymes. CYP 2D6, the isoform present in humans and monkeys, metabolizes MPTP and MPP^+^ probably to harmless compounds (Fonne-Pfister and Meyer, 1988; Jolivalt et al., 1995; Coleman et al., 1996; Gilham et al., 1997). Therefore, “CYP 2D6 poor metabolizers,” or the drugs that inhibit this isoenzyme, may represent susceptible factors favoring the neurotoxicity induced by MPTP (Barbeau et al., 1985; Lane, 1998). It is worth noting that MPP^+^ binding sites, as described by Del Zompo et al. (1986), may partly correspond in the mouse brain to the substrate recognition sites of CYP 2D isozymes. This MPP^+^ binding, indeed, is displaced potently by debrisoquine and its analogues, which are good substrates for the P450 system (Del Zompo et al., 1990). MPP^+^ binding has also been studied in post-mortem brain of PD patients and, among the several brain areas analyzed, only the Substantia Nigra (SN) showed a reduction in this binding in comparison with age-matched controls (Corsini et al., 1988). This reduction may be interpreted as a result of CYP 2D6 loss in the SN following DA neuron degeneration, a finding which is similar to that observed by Riedl et al. (1999) in the rat brain after 6-OHDA lesion of DA neurons. Furthermore, CYP 2D isoforms not only metabolize the neurotoxins MPTP and/or MPP^+^, but also markedly participate in the metabolism of methamphetamine and its analogues (Lin et al., 1995). Actually, similar conclusions must be drawn for these toxic compounds which are widely abused by humans. The role of CYP 2D6-mediated metabolism of amphetamines must be considered not only for the hepatic enzyme, but also for the one present in DA neurons. At present, it is difficult to suggest the effective physiological role of this enzyme in the DA neuron. It is likely that it behaves like a guard against endogenous or exogenous harmful intruders (false transmitters) which may affect DA metabolism. The concept of a “false transmitter” implies that endogenous chemicals may be handled within the neurons like the natural transmitter, thereby influencing the intraneuronal disposition and release of the natural transmitter (Thoenen, 1969). Among the various false transmitters which affect DA neurons, tryptamine is one of the most widely studied (Baumgarten and Zimmermann, 1992). Tryptamine is an endogenous substrate of CYP 2D6 (Martinez et al., 1997) and its involvement in PD and in schizophrenia as well has been evaluated since the 60's (Brune and Himwhich, 1962; Keuhl et al., 1968; Herkert and Keup, 1969; Smith and Kellow, 1969).

## Cytochrome P450 2E1

The highest concentration of the enzyme CYP 2E1 is in the liver where it is the main P450 enzyme for ethanol metabolism (Thomas et al., 1987; de Waziers et al., 1990; Correa et al., 2009). However, it has been found in many extrahepatic organs, for example in kidney and lung. CYP 2E1 is specifically implicated in the metabolism of several compounds including toxicants and low molecular weight procarcinogens (Koop, 1992). Among the many endogenous substrates of the enzyme that have been identified (Ronis et al., 1996; Lieber, 1997; Ingelman-Sundberg, 2004), are ketones (e.g., acetone) and fatty acids such as arachidonic acid. The exogenous compounds metabolized by CYP 2E1 comprise a wide variety of xenobiotics (e.g., acetaminophen, aniline, paracetamol, N-nitrosodimetylamine, and chlorzoxazone, with the latter two often used as enzymatic probes), alcohols (e.g., ethanol, methanol), ACE, aromatic hydrocarbons such as benzene and toluene, halogenated hydrocarbons (e.g., carbon tetrachloride) and finally anesthetics including enflurane, isoflurane, and halothane. Its active site pocket is relatively small and hydrophobic, creating a suitable environment for small non-polar molecules. Due to its existence predominantly as high spin form, CYP 2E1 also is important in oxygen reduction and generation of reactive oxyradicals, potent initiators of membrane lipid peroxidation (Ekstrom and Ingelman-Sundberg, 1989; Persson et al., 1990).

Several isoenzymes of P450 have been identified in the CNS of several animal species, including man (Kalow, 1992; Warner et al., 1993). Altough the activities of the different cytocromes in the brain are very low as compared to in the liver, CYP 2E1 has been detected by immunohistochemical techniques in the DA-containing areas, the striatum and the SN (Anandatheerthavarada et al., 1993a; Gonzalez and Kimura, 1999). As in the liver, CYP 2E1 in brain is inducible by, e.g., ethanol, isoniazid or nicotine administration (Anandatheerthavarada et al., 1993b; Sohda et al., 1993; Gonzalez and Kimura, 1999). Also, induction of this enzyme during ischemic injury was shown in hippocampal and cortex astrocytes of rat and gerbil *in vivo* (Tindberg et al., 1996; Watts et al., 1998) showed that inducible CYP 2E1 existed in the same compartment as tyrosine hydroxylase in the rat SN but could not detect the enzyme in nigral glia cells. In addition, localization of the enzyme in monkey brain, as well as prenatal and adult human brain was confirmed (Brzezinski et al., 1999; Upadhya et al., 2000; Joshi and Tyndale, 2006). The active form of CYP 2E1 has been found in ER (microsomes), in the Golgi apparatus and in the plasma membrane of rat hepatocytes (Wu and Cederbaum, 1992; Loeper et al., 1993; Neve et al., 1996). It is possible that in the CNS, the active form of this enzyme is localized in the same membrane compartments as its hepatic variety.

There is evidence that interindividual variability in the expression and functional activity of this cytochrome may be considerable. Genetic polymorphisms in CYP 2E1 were identified and linked to altered susceptibility to hepatic cirrhosis induced by ethanol and esophageal and other cancers in some epidemiological studies. Therefore, it is important to evaluate how such polymorphisms affect CYP 2E1 function and whether it is possible to construct a population distribution of CYP 2E1 activity based upon the known effects of these polymorphisms and their frequency in the population (Itoga et al., 2002; Danko and Chaschin, 2005).

Recently, considering these findings on the enzymatic properties and genetic characteristics of CYP 2E1 and the fact that the enzyme is found in the SN, preliminary data demonstrated a possible association between CYP 2E1 polymorphisms and PD (Shahabi et al., 2009).

More recently Kaut et al. (2012) found decreased methylation of the cytochrome CYP 2E1 gene and increased expression of CYP 2E1 messenger RNA in PD patients' brains, suggesting that epigenetic variants of this cytochrome contribute to PD susceptibility. Alterations of gene methylation patterns may form an interface between genetic and environmental susceptibility, carrying forward long lasting changes which may have been acquired even in preceding generations (Feinberg, 2007; Suzuki and Bird, 2008; Urdinguio et al., 2009).

Summarizing the above mentioned paragraphs the use of ACE, or other CYP 2E1 substrates/inhibitors as well, revealed the role of a specific P450 enzyme in experimental parkinsonism as obtained in the MPTP mouse model. Similarly clinical studies in PD led to the conclusion that environmental factors, such as several xenobiotics, contribute to the development of the disease. Among the relevant toxic environmental chemicals, pesticides and volatile solvents are the most suspected ones which are all substrates of CYP 2E1. It is likely that the oxidative stress induced by these substrates, including ethanol and its main metabolite ACE, may trigger a chronic impairment of DA neurons leading to degeneration. CYP 2E1 epigenetic alterations may facilitate the degenerative process through the metabolism of such xenobiotics and represent the genetic susceptibility to the disease. CYP 2E1 might be just the tip of the iceberg of epigenetic alterations to be identified in apparently sporadic neurodegenerative disorders.

### Conflict of interest statement

The authors declare that the research was conducted in the absence of any commercial or financial relationships that could be construed as a potential conflict of interest.
